# Comparison of short-term clinical efficacy between modified Kamikawa anastomosis and double tract anastomosis after laparoscopic proximal gastrectomy

**DOI:** 10.3389/fonc.2024.1414120

**Published:** 2024-09-02

**Authors:** Chu-Ying Wu, Qiao-Zhen Huang, Kai Ye

**Affiliations:** Department of Gastrointestinal Surgery, The Second Affiliated Hospital of Fujian Medical University, Quanzhou, China

**Keywords:** adenocarcinoma of esophagogastric junction, upper gastric adenocarcinoma, proximal gastrectomy, Kamikawa anastomosis, double tract anastomosis

## Abstract

**Objective:**

This study aimed to explore the short-term clinical efficacy of modified Kamikawa anastomosis and double tract anastomosis after laparoscopic proximal gastrectomy.

**Methods:**

A retrospective analysis was carried out by collecting the clinical and pathological data of 42 patients who underwent laparoscopic proximal gastrectomy after admission in our centre from May 2020 to October 2022. Among the 42 enrolled patients, 22 underwent modified Kamikawa anastomosis (modified Kamikawa group), and the other 20 underwent double tract anastomosis (double tract group). Outcome measures included intraoperative condition, postoperative recovery, postoperative quality of life, postoperative nutritional status and gastroesophageal reflux. The patients were followed up using outpatient examination and telephone interviews to identify their nutritional status, reflux esophagitis and anastomotic status.

**Results:**

(1) Intraoperative condition: Compared with the double tract group, the modified Kamikawa group had significantly prolonged time for operation and digestive tract reconstruction. However, no statistically significant difference in intraoperative blood loss was found between the two groups. (2) Postoperative recovery: Compared with the double tract group, the modified Kamikawa group had a significantly shorter time for the first postoperative intake of fluids, drainage tube placement and postoperative hospital stay. No statistically significant difference in the time to first postoperative anal exhaust and postoperative complications was found between the two groups. (3) Postoperative quality of life: Compared with the double tract group, the modified Kamikawa group showed better quality of life at 12 months after surgery. (4) Postoperative nutritional status and gastroesophageal reflux: No statistically significant difference in hemoglobin, total serum albumin, albumin, body mass index, MUST score, PG-SGA score, grading of reflux esophagitis using the Los Angeles classification system and GERD score was found between the two groups at 6 and 12 months after surgery. All patients did not experience anastomotic stenosis and tumour recurrence or metastasis.

**Conclusions:**

Modified Kamikawa anastomosis is a safe and feasible treatment in laparoscopic proximal gastrectomy, which can ensure good postoperative anti-reflux effect and nutritional status. It has the advantage of better postoperative recorvery and quality of life compared with double tract anastomosis.

## 1 Introduction

Adenocarcinoma of the esophagogastric junction is characterised by a continuous rise in incidence and a relatively high incidence rate in early gastric cancer worldwide (1–4). Special attention has been paid to function-preserving gastric surgery to further improve the postoperative quality of life of patients. Compared with total gastrectomy, proximal gastrectomy leads to a lower incidence of complications, better nutritional status and less weight loss among patients (5–7). Therefore, proximal gastrectomy for tumours at the esophagogastric junction and in the upper part of the stomach has become a hotspot in clinical research. However, this procedure may cause damage to the lower oesophageal sphincter, resulting in the loss of the original cardiac function of anti-reflux. Additionally, postoperative vagus nerve injury may reduce the compliance of the gastric remnant, leading to the evacuation disorder of the gastric remnant and, thus, the exacerbation of gastroesophageal reflux (8, 9). Interstitial jejunal double tract anastomosis has a good anti-reflux effect and low requirement for the size of the gastric remnant and can be applied for digestive tract reconstruction after proximal gastrectomy. Kamikawa anastomosis, a recently emerging technique, has become a research highlight for digestive tract reconstruction after proximal gastrectomy due to its excellent anti-reflux effect (10). Our centre has modified the traditional Kamikawa anastomosis procedure to reduce the operational difficulty and shorten the operative time while ensuring the anti-reflux effect and radical tumour resection. This study retrospectively analysed the clinical and pathological data of 42 patients who had adenocarcinoma of esophagogastric junction and upper gastric adenocarcinoma and underwent laparoscopic proximal gastrectomy after admission to our centre from May 2020 to October 2022. This research aims to investigate the short-term clinical efficacy of modified Kamikawa anastomosis and double tract anastomosis.

## 2 Materials and methods

### 2.1 General data

A retrospective analysis was carried out by collecting the clinical and pathological data of 42 patients (34 males and 8 females; 40-83 years old) who had adenocarcinoma of esophagogastric junction and upper gastric adenocarcinoma and underwent laparoscopic proximal gastrectomy after admission in our centre from May 2020 to October 2022. Among the enrolled 42 patients, 22 underwent laparoscopic proximal gastrectomy and modified Kamikawa anastomosis (modified Kamikawa group), and the other 20 received laparoscopic proximal gastrectomy and double tract anastomosis (double tract group).

### 2.2 Inclusion and exclusion criteria

Inclusion criteria were as follows: (1) patients with confirmed adenocarcinoma of esophagogastric junction and upper gastric adenocarcinoma via preoperative pathological examination through gastroscopy; (2) patients with tumour diameter of <4 cm; (3) esophageal infiltration length is less than 2cm; (4) patients with clinical stage of cT1-2N0M0 via preoperative enhanced CT, ultrasonic gastroscopy; (5) patients without distant metastasis before surgery; and (6) patients without a history of abdominal surgery.

Exclusion criteria were as follows: (1) patients with preoperative neoadjuvant treatment; (2) patients with severe cardiopulmonary dysfunction and poor nutritional status who had difficulty tolerating surgery; (3) patients with other malignant tumours; and (4) patients with incomplete clinical and pathological data.

### 2.3 Surgical procedures

All surgeries in this study were completed by the same team of surgeons. Under intravenous inhalation combined with general anaesthesia, the patients were placed in a supine split-leg position, with the head slightly elevated. The surgical site was prepped and draped in the usual sterile fashion after successful anaesthesia administration. The surgeon operated on the left side of the patient, the assistant assisted on the right side of the patient, and another assistant who supported the laparoscopy stood between the patient’s legs. Following the five-port method, a 12 mm Trocar was placed in the infra-umbilical region (the observation port) to establish a pneumoperitoneum, and the pressure was maintained at 12-15 mmHg(1 mmHg=0.133 kPa). Afterwards, a 12 mm Trocar and a 5 mm Trocar were placed 2 cm below the costal margin at the left anterior axillary line and 2 cm above the level of the umbilicus at the left midclavicular line, respectively (operating ports). Contralateral operating ports were also established by inserting a 5 mm Trocar at the corresponding position on the right side. Proximal gastric dissociation and D1+ lymph node dissection were performed after the laparoscopic exploration of the tumour’s location, size, infiltration degree and relationship with surrounding organs and tissues.

#### 2.3.1 Double tract group

**Figure 1:**
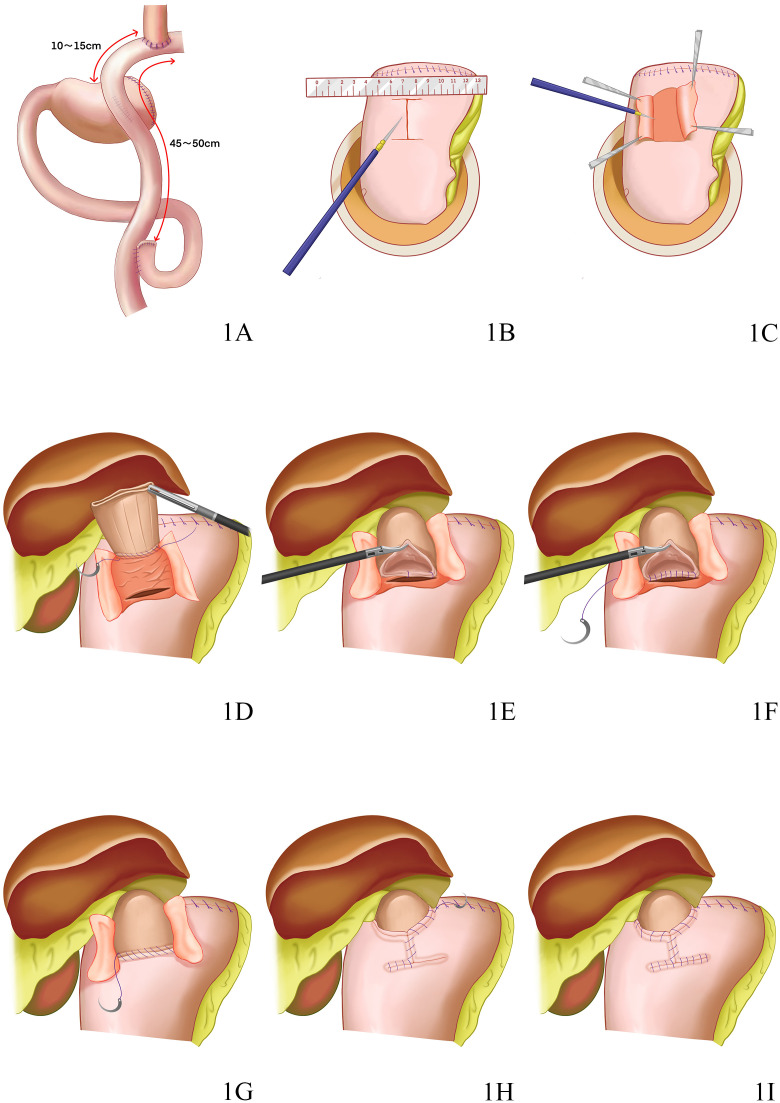
**(A)** The reconstruction after dual channel anastomosis is shown. **(B)** An ‘I’-shaped mark [(2.5-3) cm × 3.5 cm] was marked with gentian violet on the anterior wall of the gastric remnant (approximately 1.5 cm from the upper incisal margin) near the lesser curvature of stomach to match the width of the seromuscular flap with the oesophageal diameter; the upper edge of the seromuscular flap must be parallel to the incisal margin of the upper end of the gastric remnant. **(C)** The muscle flap should be vertically pulled upwards by the assistant to create tension, followed by separation by the surgeon using an electrotome to ensure the complete separation of the submucosa and muscle layer. **(D)** The lower segment of the oesophagus, marked with gentian violet, was continuously sutured and fixed to the upper edge of the seromuscular flap using a 3-0 5/8 curved endoscopic suture. **(E)** The posterior wall of the oesophageal stump opening and the upper edge of the anastomotic stoma were intermittently sutured with two sutures on the left and middle for fixation. **(F)** The entire layer of the posterior wall of the oesophageal stump was then continuously sutured with the gastric mucosa and submucosa at the upper edge of the anastomotic stoma using a 3-0 barbed suture from left to right until the right edge. **(G)** The entire anterior wall of the oesophageal stump was then continuously sutured with the entire stomach layer at the lower edge of the anastomotic stoma using another 3-0 barbed suture from left to right until the right edge. **(H)** Another suture from the right side to the intersection point of bilateral seromuscular flaps was placed using the reserved barbed suture, followed by sutures for the seromuscular flap at the anastomotic stoma and the left oesophageal ‘Y’-shaped edge upwards. **(I)** While the anastomotic stoma was being covered, the reconstruction of modified Kamikawa anastomsis was completed.

The tumour and proximal stomach were removed after the transection of the oesophagus. The jejunum and mesenteric blood vessels were cut off at a distance of 20-25 cm from the suspensory ligament of the duodenum. The oesophagus was then anastomosed to the distal jejunum, and the broken end of the jejunum was closed with the linear cutter stapler, with the cecum measuring 2-3 cm in length. Proximal-distal jejunal anastomosis was performed at a distance of 45-50 cm from the distal end of the oesophagojejunal anastomosis to complete the Roux-en-Y oesophagojejunal anastomosis. Another side-to-side anastomosis of the jejunum and anterior wall of the gastric remnant was conducted at a distance of 10-15 cm from the oesophagojejunal anastomosis to close the gastric stump. For this process, the use of a 60 mm linear cutter stapler for Roux-en-Y anastomosis was recommended to expand the gastrointestinal anastomotic stoma and facilitate food passage. Finally, the dual channel was established (**Figure 1A**).

#### 2.3.2 Modified Kamikawa group

This procedure was started with the cropping of the falciform ligament, separation of the left triangular ligament and part of the lesser omentum and dissociation of the left lateral lobe of the liver. The left lateral lobe was then lifted upwards and placed on the right lobe of the liver after passing the cropped falciform ligament. Meanwhile, the liver was fixed and suspended with the assistance of a suture needle and suture for external purse-string suture. The tumour was intraoperatively localised under gastroscopy. After the oesophageal hiatus was opened, the oesophagus was fully dissociated and dissected, and the posterior wall 5 cm away from the oesophageal stump was marked with gentian violet. A small subxiphoid incision was made to pull out the stomach, and the proximal stomach was severed with the linear cutter stapler at a distance of 3 cm from the distal end of the tumour, followed by the interrupted suture of the gastric stump for reinforcement. Intraoperative rapid pathological examination of the frozen section of the collected incisal margin was performed as needed. An ‘I’-shaped mark [(2.5-3) cm × 3.5 cm] was marked with gentian violet on the anterior wall of the gastric remnant (approximately 1.5 cm from the upper incisal margin) near the lesser curvature of stomach to match the width of the seromuscular flap with the oesophageal diameter; the upper edge of the seromuscular flap must be parallel to the incisal margin of the upper end of the gastric remnant (**Figure 1B**). The submucosal layer was dissected and dissociated from the muscular layer along the mark to prepare the seromuscular flap while protecting the integrity of the seromuscular flap and gastric mucosa. At this point, the muscle flap should be vertically pulled upwards by the assistant to create tension, followed by separation by the surgeon using an electrotome to ensure the complete separation of the submucosa and muscle layer (**Figure 1C**). The submucosal layer and mucosal layer were cut open at the lower edge of the seromuscular flap for anastomosis; the cut width must be equivalent to that of the oesophagus. The gastric remnant was then placed into the abdominal cavity to establish a pneumoperitoneum repeatedly. Following the traction of the oesophagus under laparoscopy, the right wall of the lower oesophageal stump was cut open using an ultrasonic knife and extended outside the gastric tube as guidance. The lower segment of the oesophagus, marked with gentian violet, was continuously sutured and fixed to the upper edge of the seromuscular flap using a 3-0 5/8 curved endoscopic suture (**Figure 1D**). After the oesophageal stump was opened with an ultrasound knife, the posterior wall of the oesophageal stump opening and the upper edge of the anastomotic stoma were intermittently sutured with two sutures on the left and middle for fixation (**Figure 1E**). The next step was the continuous suture of the entire layer of the posterior wall of the oesophageal stump with the gastric mucosa and submucosa at the upper edge of the anastomotic stoma using a 3-0 barbed suture from left to right until the right edge (**Figure 1F**). The suture needle was simultaneously threaded through the mucosa of the anterior wall of the oesophagus and out of the outer membrane for the subsequent suture of the seromuscular flap. The entire anterior wall of the oesophageal stump was then continuously sutured with the entire stomach layer at the lower edge of the anastomotic stoma using another 3-0 barbed suture from left to right until the right edge (**Figure 1G**). The reserved barbed suture was threaded through the serosal layer at the lower right corner of the seromuscular flap. The lower ends of bilateral seromuscular flaps were crossed and fixed on the anterior wall of the stomach below the midpoint of the anastomotic stoma. Another suture from the right side to the intersection point of bilateral seromuscular flaps was placed using the reserved barbed suture, followed by sutures for the seromuscular flap at the anastomotic stoma and the left oesophageal ‘Y’-shaped edge upwards (**Figure 1H**). Another barbed suture was used to suture the seromuscular flap at the anastomotic stoma and the right oesophageal ‘Y’-shaped edge; while the anastomotic stoma was being covered, the reconstruction of modified Kamikawa anastomsis was completed (**Figure 1I**). Finally, the condition of the anastomotic stoma was examined under gastroscopy to observe the presence of any stenosis at the stoma.

### 2.4 Outcome measures and evaluation criteria

The outcome measures were as follows: (1) intraoperative condition, which involves surgical condition, operative time, intraoperative blood loss and digestive tract reconstruction time; (2) postoperative recovery, which includes time to first postoperative anal exhaust, time to first postoperative intake of fluids, drainage tube placement time, postoperative hospital stay, postoperative complications (intestinal obstruction, lymphatic fistula, abdominal bleeding, anastomotic bleeding, anastomotic fistula, gastroparesis and pulmonary infection) and postoperative complication grading; (3) postoperative quality of life and (4) postoperative nutritional status and gastroesophageal reflux, which includes the number of patients receiving follow-up, follow-up time, nutritional status after discharge, reflux esophagitis and anastomotic status.

The evaluation criteria were as follows: the postgastrectomy syndrome assessment scale (PGSAS-45) designed by the Japanese Postgastrectomy Syndrome Working Party (JPGSWP) was used to determine the intensity of various symptoms after gastrectomy and their impact on patients’ quality of life. The scale mainly consists of symptoms, living status and quality of life domains. Related problems in different domains were graded based on their severity. High scores on the subscales of body mass change, food intake per meal and meal quality and the total scores of physical health and mental health indicated a good condition; for the other items, high scores suggested a poor condition (11). Postoperative complications were graded using the Clavien-Dindo classification (11). Nutritional status was assessed based on hemoglobin, total serum albumin, albumin, BMI, Malnutrition Universal Screening Tool score (MUST score) and Patient-Generated Subjective Global Assessment (PG-SGA) score (12). Symptoms of oesophageal reflux were scored using the Gastroesophageal Reflux Disease (GERD) scale (13). Anastomotic stenosis is defined as a condition where the diameter of the anastomosis is less than 1cm when observed under endoscopy, or when an ordinary gastroscope is unable to pass through. Diagnosis of reflux esophagitis adopted gastroscopy, and evaluation of the degree of lesion employed the Los Angeles classification system (14). Additionally, anastomotic status was identified using upper digestive tract radiography.

### 2.5 Follow-up

The follow-up of patients after discharge was planned to be at least 12 months, which ended in October 2023. The patients were followed up via outpatient examination and telephone interview to determine their quality of life, specific nutritional status, reflux esophagitis and anastomotic status. All patients underwent gastroscopy and upper digestive tract angiography one year after the surgery.

### 2.6 Statistical analysis

Measurement data with normal distribution and skewed distribution were represented by x ± s and M (range), respectively. T-test and Mann-Whitney U test were used for intergroup comparison, and the non-parametric rank sum test was utilised for ranked data comparison. Counting data were expressed in absolute numbers, and intergroup comparison adopted χ² test or Fisher’s Exact Test. P<0.05 indicated a statistically significant difference.

## 3 Results

### 3.1 Clinicopathological characteristics

No statistically significant difference in gender, age, body mass index (BMI), tumour location, maximum tumour diameter, tumour differentiation degree and pathological staging between the two groups (P>0.05; **Table 1**), suggesting comparability.

**Table 1:** Clinicopathological characteristics.

	Modified Kamikawa group	Double tract group	P value
Number	22	20	
Sex			1.000
Male	18	16	
Female	4	4	
Age (x ± s, years)	68.3 ± 6.6	67.5 ± 10.0	0.754
BMI (x ± s, kg/m^2^)	22.2 ± 2.9	21.2 ± 3.3	0.313
Tumour location			0.119
Esophagogastric junction	3	3	
Cardia of stomach	16	9	
Fundus of stomach	3	8	
Maximum tumour diameter (M (range), cm)	1.9 (0.7-3.8)	3.0 (0.5-4.0)	0.122
Histological grade			0.113
Poor	7	10	
Moderately	7	8	
Well	8	2	
Tumor stage			0.118
I	18	12	
II	4	8	

### 3.2 Intraoperative condition

Laparoscopic proximal gastrectomy combined with D1 + lymph node dissection was completed successfully in all the patients. Statistically significant differences in time for operation [210.0 (150.0-240.0) min vs. 186.5 (167.0-226.0) min] and digestive tract reconstruction [89.0 (78.0-116.0) min vs. 80.0 (73.0-97.0) min] were observed between the two groups (both P<0.05). No statistically significant difference in intraoperative blood loss was found between the two groups (21.0 ± 3.8 mL vs. 22.9 ± 4.7 mL; P>0.05). The above data are presented in **Table 2**.

**Table 2:** Operation conditions.

	Modified Kamikawa group	Double tract group	P value
Number	22	20	
The operation time (M (range), min)	210.0 (150.0-240.0)	186.5 (167.0-226.0)	0.049
The digestive tract reconstruction time (M (range), min)	89.0 (78.0-116.0)	80.0 (73.0-97.0)	0.033
The intraoperative blood loss (x ± s, ml)	21.0 ± 3.8	22.9 ± 4.7	0.167

### 3.3 Postoperative recovery

Statistically significant differences in the time to first postoperative intake of fluids [3.5 (3.0-5.0) d vs. 4.0 (3.0-6.0) d], drainage tube placement time [6.5 (5.0-8.0) d vs. 8.0 (6.0-9.0) d] and postoperative hospital stay [8.0 (7.0-10.0) d vs. 9.00 (8.0-11.0) d] were observed between the two groups (all P<0.05). No statistically significant difference in the time to first postoperative anal exhaust was found between the two groups [2.0 (1.0-3.0) d vs. 2.0 (1.0-4.0) d; P>0.05]. Postoperative intestinal obstruction, anastomotic stenosis, anastomotic hemorrhage, anastomotic fistula and pulmonary infection were found in 0, 0, 0, 0 and 1 patient in the modified Kamikawa group, respectively, and in 0, 0, 1, 0 and 1 patient in the double tract group, respectively; no statistically significant differences were observed between the two groups (all P>0.05). Postoperative complications classified as Clavien-Dindo grade 1, 2, 3 and 4 were found in 0, 1, 0 and 0 patient(s) in the modified Kamikawa group, respectively, and in 1, 1, 0 and 0 patient(s) in the double tract group, respectively; no statistically significant difference was observed between the two groups (all P>0.05). All postoperative complications in both groups were improved after conservative treatment. The above data are presented in **Table 3**.

**Table 3:** Postoperative recovery.

	Modified Kamikawa group	Double tract group	P value
Number	22	20	
The time of first postoperative anal exhaust (M (range), d)	2.0 (1.0-3.0)	2.0 (1.0-4.0)	0.070
The time of first postoperative fluid intake (M (range), d)	3.5 (3.0-5.0)	4.0 (3.0-6.0)	0.042
The time of drainage tube placement time (M (range), d)	6.5 (5.0-8.0)	8.0 (6.0-9.0)	0.000
The time of postoperative stay (M (range), d)	8.0 (7.0-10.0)	9.00 (8.0-11.0)	0.026
Postoperative complications			0.932
Intestinal obstruction	0	0	
Anastomotic stenosis	0	0	
Anastomotic hemorrhage	0	1	
Anastomotic fistula	0	0	
Pulmonary infection	1	1	
Clavien-Dindo classification			1.000
1	0	1	
2	1	1	
3	0	0	
4	0	0	

### 3.4 Postoperative quality of life

Compared with the double tract group, the modified Kamikawa group exhibited better gastroesophageal reflux (3.1 ± 1.3 vs. 4.0 ± 1.3), eating discomfort (2.9 ± 1.0 vs. 3.7 ± 1.0) and total symptom score (2.9 ± 1.1 vs. 3.8 ± 1.2) in the physical symptom domain, quality of ingestion [3.7 (3.0, 5.7) vs. 3.3 (3.0, 5.0)] in the living status domain, postoperative symptoms [1.0 (1.0, 3.0) vs. 2.0 (1.0, 3.0)], meals and daily lives [3.7 (3.0, 5.7) vs. 3.3 (3.0, 5.0); and 1.7 (1.0, 3.8) vs. 2.3 (1.0, 3.7)] in the quality of life domain 12 months after surgery, and the differences were statistically significant (all P<0.05). The above data are presented in **Table 4**.

**Table 4:** Postoperative quality of life.

	Modified Kamikawa anastomosis group	Double-tract anastomosis group	P value
Number	22	20	
Symptoms			
Esophageal reflux subscale (x ± s)	3.1 ± 1.3	4.0 ± 1.3	0.032
Abdominal pain subscale (M (range))	1.7 (1.3, 4.3)	2.0 (1.3, 4.3)	0.241
Meal-related distress subscale (x ± s)	2.9 ± 1.0	3.7 ± 1.0	0.020
Indigestion subscale (M (range))	2.4 (2.0, 4.0)	2.5 (2.0, 4.8)	0.828
Diarrhea subscale (M (range))	1.3 (1.0, 2.7)	1.5 (1.0, 2.7)	0.630
Constipation subscale (M (range))	1.3 (1.0, 2.3)	1.3 (1.0, 2.3)	0.325
Dumping subscale (M (range))	1.3 (1.0, 23)	1.3 (1.0, 2.3)	0.580
Total symptom score (x ± s)	2.9 ± 1.1	3.8 ± 1.2	0.025
Other outcome measures (symptom)			
Increased flatus (M (range))	3.0 (1.0, 6.0)	3.5 (1.0, 6.0)	0.091
Loose stools (M (range))	1.0 (1.0, 2.0)	1.0 (1.0, 2.0)	0.865
Living status			
Change in body weight (%)^a^ (x ± s)	12.6 ± 4.6	13.8 ± 5.1	0.444
Ingested amount of food per meal^a^ (M (range))	4.0 (4.0, 6.0)	4.0 (4.0, 6.0)	0.383
Necessity for additional meals (M (range))	3.0 (3.0, 5.0)	3.5 (3.0, 5.0)	0.173
Quality of ingestion subscale^a^ (M (range))	3.7 (3.0, 5.7)	3.3 (3.0, 5.0)	0.048
Ability for working^a^ (M (range))	1.0 (1.0, 2.0)	1.0 (1.0, 2.0)	0.597
Quality of life			
Dissatisfaction with symptoms (M (range))	1.0 (1.0, 3.0)	2.0 (1.0, 3.0)	0.003
Dissatisfaction at the meal (M (range))	1.0 (1.0, 3.0)	2.0 (1.0, 3.0)	0.000
Dissatisfaction at working (M (range))	1.0 (1.0, 2.0)	1.0 (1.0, 2.0)	0.221
Dissatisfaction for daily life subscale (M (range))	1.7 (1.0, 3.8)	2.3 (1.0, 3.7)	0.043
Physical component summary^a^ (x ± s)	80.5 ± 5.6	80.8 ± 5.2	0.838
Mental component summary^a^ (x ± s)	89.8 ± 6.1	90.0 ± 5.6	0.923

In items or subscales with ^a^, higher score indicates better condition. In items or subscales without ^a^, higher score indicates worse condition.

### 3.5 Postoperative nutritional status and gastroesophageal reflux

All patients were followed up for 6-12 months after surgery. There was no significant difference in BMI scores before and after surgery in either group (P>0.05). No statistically significant difference in hemoglobin (124.5 ± 15.4 g/L vs. 122.0 ± 21.1 g/L and 125.8 ± 13.5 g/L vs. 125.4 ± 16.8 g/L), total serum albumin (69.1 ± 4.8 g/L vs. 67.2 ± 4.5 g/L and 69.5 ± 4.1 g/L vs. 68.5 ± 4.6 g/L), albumin (43.1 ± 3.7 g/L vs. 42.1 ± 3.2 g/L and 43.4 ± 2.8 g/L vs. 43.1 ± 2.6 g/L), BMI (22.2 ± 2.7 kg/m^2^ vs. 22.8 ± 2.9 kg/m^2^ and 22.4 ± 2.5 kg/m^2^ vs. 21.5 ± 2.9 kg/m^2^), MUST score [1.0 (1.0-2.0) vs. 1.0 (1.0-2.0) and 12 (1-2) vs. 1.0 (1.0-2.0)], PG-SGA score [2.0 (1.0-3.0) vs. 2.0 (1.0-3.0 and 2.0 (2.0-3.0) vs. 1.5 (1.0-3.0)], GERD scale score [3.0 (2.0-4.0) vs. 3.0 (2.0-4.0) and 3.0 (2.0-4.0) vs. 2.5 (2.0-4.0)] and cases with ≥Grade B reflux esophagitis (N = 1 vs. N = 2) was found between the two groups at 6 and 12 months after surgery (all P>0.05). All patients in both groups did not experience anastomotic stenosis. The above data are presented in **Table 5**.

**Table 5:** Postoperative nutritionnal status and gastroesophageal reflux.

	Modified Kamikawa group	Double tract group	P value
Number	22	20	
Hemoglobin 6 months after surgery (x ± s, g/L)	124.5 ± 15.4	122.0 ± 21.1	0.649
Hemoglobin 12 months after surgery (x ± s, g/L)	125.8 ± 13.5	125.4 ± 16.8	0.937
Total serum albumin 6 months after surgery (x ± s, g/L)	69.1 ± 4.8	67.2 ± 4.5	0.194
Total serum albumin 12 months after surgery (x ± s, g/L)	69.5 ± 4.1	68.5 ± 4.6	0.455
Albumin 6 months after surgery (x ± s, g/L)	43.1 ± 3.7	42.1 ± 3.2	0.383
Albumin 12 months after surgery (x ± s, g/L)	43.4 ± 2.8	43.1 ± 2.6	0.752
BMI 6 months after surgery (x ± s, kg/m^2^)	22.2 ± 2.7	22.8 ± 2.9	0.111
BMI 12 months after surgery (x ± s, kg/m^2^)	22.4 ± 2.5	21.5 ± 2.9	0.245
MUST score 6 months after surgery (M (range))	1.0 (1.0-2.0)	1.0 (1.0-2.0)	0.231
MUST score 12 months after surgery (M (range))	1.0 (1.0-2.0)	1.0 (1.0-2.0)	0.064
PG-SGA score 6 months after surgery (M (range))	2.0 (1.0-3.0)	2.0 (1.0-3.0)	0.434
PG-SGA score 12 months after surgery (M (range))	2.0 (1.0-3.0)	1.5 (1.0-3.0)	0.585
GERD scale score 6 months after surgery (M (range))	3.0 (2.0-4.0)	3.0 (2.0-4.0)	0.690
GERD scale score 12 months after surgery (M (range))	3.0 (2.0-4.0)	2.5 (2.0-4.0)	0.671
≥Grade B reflux esophagitis	1	2	0.870

## 4 Discussion

The gradual increase in the incidence of upper gastric cancer has attracted increasing attention from surgeons (15). A national survey in South Korea reported that the incidence of proximal gastric cancer increased from 11.2% in the past to 16% in 2014 (16). Total gastrectomy is the traditional therapeutic option for the treatment of upper gastric cancer. With the development of modern medicine and the improvement in the detection of early gastric cancer, preserving gastric function to the greatest extent while ensuring complete radical tumour resection has become a new clinical demand to cope with the trend of the times. This goal promotes the emergence of function-preserving proximal gastrectomy. For patients with early gastric cancer, proximal gastrectomy can effectively improve their postoperative nutritional status and has no influence on their long-term survival. However, reflux esophagitis may occur in up to 21.8%—71.6% of patients undergoing traditional esophagogastric anastomosis, which seriously affects their postoperative quality of life (17). Procedures such as tubular gastroesophageal anastomosis, side-to-side stapled esophagogastric anastomosis, double tract anastomosis and anastomosis have been developed for digestive tract reconstruction after proximal gastrectomy (18). Despite the effectively reduced occurrence of postoperative reflux, the risks of operational difficulties or anastomotic complications arise to some extent (19). Therefore, domestic and international research focused on how to achieve surgical safety and ensure postoperative quality of life in digestive tract reconstruction after proximal gastrectomy.

For the first time, Aikou et al. (20) in Japan (1988) reported that double tract anastomosis had a good anti-reflux effect and was applicable for digestive tract reconstruction in the vast majority of patients undergoing proximal gastrectomy, especially for patients with small gastric remnant; however, it was unsuitable for those with oesophagus-gastric remnant anastomosis and reduced glucose tolerance. Nakajima et al. (21) found that a large gastric remnant was suitable for the transport and mixing of bile and food for patients undergoing double tract anastomosis after proximal gastrectomy; moreover, a small portion of food directly entering the jejunum could alleviate the slow emptying or accumulation of food in the gastric remnant caused by vagotomy. By conducting routine gastric emptying scans 3 months after surgery, Ahn et al. (8) reported an average gastric emptying time of 164.3 min, indicating a delay in gastric emptying to some extent; the relative ratio of food flow between the stomach and small intestine was 3:2 after double tract anastomosis. In some cases, food may not always enter the stomach and duodenum smoothly as expected after double tract anastomosis and instead directly enters the distal jejunum; additionally, the procedure may not be beneficial to patients if the ingested food cannot pass through the gastric remnant (22, 23). Owing to the complicated operation and the possibility of forming many anastomotic stomas, double tract anastomosis may be associated with a high risk of postoperative anastomotic leakage, accompanied by the increased cost attributed to the use of additional linear cutter staplers.

In 1998, Kamikawa (24) reported a new type of double-flap technique for digestive tract reconstruction, also known as Kamikawa anastomosis. Its indications include patients with upper gastric cancer and an estimated volume of postoperative gastric remnant of >50%. Kamikawa anastomosis has gained popularity due to its excellent anti-reflux effect and low risk of postoperative anastomotic leakage. Muraoka et al. (25) applied Kamikawa anastomosis for the first time in laparoscopic proximal gastrectomy in 2016 and were not able to observe postoperative reflux esophagitis in all the patients. Kuroda et al. (10) also reported the application of Kamikawa anastomosis after laparoscopic proximal gastrectomy, confirming its clinical efficacy, safety and feasibility. In this procedure, a seromuscular flap is prepared to cover the lower oesophageal segment and the stoma. Similar to a reconstructed cardia, this flap acts as a one-way valve to increase the pressure at these two sites, thereby achieving an anti-reflux effect. The risk of postoperative anastomotic leakage might be low because of the presence of only one anastomotic stoma that is covered by the seromuscular flap after digestive tract reconstruction. Meanwhile, Shoji et al. (26) reported that the application of Kamikawa anastomosis in proximal gastrectomy might lead to a low incidence of postoperative anastomotic complications and effectively reduce the occurrence of reflux esophagitis. In another multicentre retrospective study of 464 cases in Japan, only 6% of cases with reflux esophagitis were found during gastroscopy 1 year after proximal gastrectomy and Kamikawa anastomosis (24). The anastomotic stoma used for Kamikawa anastomosis is generally made by manual suture and thus entails a low cost. However, its complicated process necessitates high requirements for the surgeon’s operation, especially the suture technique under endoscopy, which is significantly more time-consuming compared with other procedures used for digestive tract reconstruction. In addition, the improper preparation of the seromuscular flap may lead to ischaemia of the flap and the stenosis of the stoma. As a consequence, the popularisation of this surgical technique is restricted to a certain extent. In the present study, the traditional Kamikawa anastomosis was modified to some extent while ensuring the anti-reflux effect and complete radical tumour resection.

The following points are main modifications of original Kamikawa anastomosis performed by the author’s centre. (1) The left extrahepatic lobe is separated and suspended, thus, reducing the blocking and ensuring a good view and easy operation. Additionally, the operation requires no extra instrument, is simple and practicable and does not cause any trauma to the liver. (2) As the width of the esophagus is commonly 2.5-3 cm, the width of the I-shaped seromuscular flap is changed to 2.5-3 cm to match the diameter of the esophagus and consequently lower the incidence of anastomotic stenosis. (3) The posterior wall of the esophageal stump opening and the superior border of the anastomotic stoma are firstly fixed by placing two interrupted sutures on the right and in the middle. When the posterior wall of the esophageal stump opening and the superior border of the anastomotic stoma are fixed, subsequent sutures are easier and less likely to shift and cause postoperative anastomotic stenosis. (4) Added the use of continuous sutures, including the suture of the posterior wall of the esophageal stump, the anastomotic stoma, and the seromuscular flap, to make the operation smoother, thus avoiding the tedious operation of interrupted suture and reducing the duration of the operation. (5) After proximal gastric resection, the blood supply to the incisal margin is poor. If the seromuscular muscle flap is close to the incisal margin, it may lead to postoperative ischemia and affect the anti-reflux effect. In our operation, the superior border of the I-shaped seromuscular flap is parallel to the upper incisal edge of the gastric remnant to ensure that the seromuscular flap is close to the lesser curvature side of the stomach with better blood supply, thus improving the blood supply in the seromuscular flap and allowing it to perform the anti-reflux effect. (6) His Angle is formed after traditional Kamikawa anastomosis, while the pseudofornix reconstructed by our modified method on the left side of the esophagus is larger and has better anti-reflux effect. Attention should be paid to the tension of the seromuscular flap. When the tension is too large during the folded suture of the seromuscular flap, the seromuscular flap may be directly sutured obliquely to the esophageal wall to reduce the tension and prevent postoperative anastomotic stenosis. With the modifications we’ve made, the technology has become more feasible than traditional operation. Compared with the traditional approach, the improved Kamikawa anastomosis in this study can reduce the duration of operation to about half (27).

In this study, the patients who underwent modified Kamikawa anastomosis had significantly shorter time for the first postoperative intake of fluids, drainage tube placement time and postoperative hospital stay compared with those who underwent double tract anastomosis. However, the former procedure resulted in longer operative time and digestive tract reconstruction time than the latter due to the complexity and difficulty of the surgery. Nevertheless, the time for anastomosis may be shortened if the surgical team cooperates smoothly and continuously optimises the surgical process. The surgery is recommended for patients with tumours that do not invade the dentate line in the early stage to reduce the difficulty of surgery and ensure surgical safety. Furthermore, given their short time for the first postoperative intake of fluids, drainage tube placement time and postoperative hospital stay, the patients who underwent modified Kamikawa anastomosis can be considered to have a fast postoperative recovery due to the preservation of relatively normal digestive tract structure and few anastomoses.

Innovations of the present study include a systematic evaluation of quality of life after proximal gastrectomy with modified Kamimawa anastomosis versus double tract anastomosis. Compared with the previous studies, the PGSAS-45 scale designed by the JPGSWP was used in the present study to evaluate the postoperative quality of life of patients in the two groups. This scale is the only comprehensive questionnaire suitable for patient assessment after different gastrectomy and reconstruction operations (28). The results of the present study show that in the physical symptom domain, gastroesophageal reflux symptoms and eating discomfort 12 months after surgery were improved in the modified Kamimawa group compared with those in the double tract group. In the postoperative quality of life domain, the modified Kamimawa group showed increased satisfaction with daily living compared with that in the double tract group. During the follow-up period, gastroesophageal reflux and eating discomfort considerably affected the life of patients, and modified Kamimawa effectively reduced the development of these symptoms. These results indicate that the overall postoperative quality of life of patients was increased in the modified Kamimawa group compared with that in the double tract group.

In addition, there were no differences in the nutritional status and reflux esophagitis of the two groups of patients during the follow-up, indicating that both methods can achieve good anti-reflux effects while ensuring food intake. Despite the availability of various procedures for digestive tract reconstruction, the following basic principles must be met: (1) reducing the incidence of anastomotic complications; (2) meeting the functional needs of patients after surgery; and (3) providing benefits during the postoperative follow-up of patients. Modified Kamikawa anastomosis meets the above principles and is expected to become one of the options for digestive tract reconstruction after proximal gastrectomy.

In summary, modified Kamikawa anastomosis and double tract anastomosis after laparoscopic proximal gastrectomy is a safe and feasible treatment for patients with adenocarcinoma of esophagogastric junction and upper gastric adenocarcinoma, both of which can ensure good postoperative anti-reflux effect and nutritional status. Despite its longer operative time, modified Kamikawa anastomosis after laparoscopic proximal gastrectomy has the advantages of postoperative recorvery and quality of life compared with double tract anastomosis. This study was designed as a retrospective analysis with small sample size. Further validation with a large sample size and long follow-up must be conducted in the future.

## Data Availability

The raw data supporting the conclusions of this article will be made available by the authors, without undue reservation.
